# Actin polymerisation at the cytoplasmic face of eukaryotic nuclei

**DOI:** 10.1186/1471-2121-7-23

**Published:** 2006-05-23

**Authors:** Sylvia Münter, Jost Enninga, Rafael Vazquez-Martinez, Erwan Delbarre, Brigitte David-Watine, Ulf Nehrbass, Spencer L Shorte

**Affiliations:** 1 Unité de Biologie Cellulaire du Noyau, CNRS URA 2582, Département de Biologie Cellulaire et Infection, Institut Pasteur; 25, Rue du Docteur Roux, 75724 Paris Cedex 15, France; 2 Current address: Department of Parasitology, Heidelberg University School of Medicine, Im Neuenheimer Feld 326, 69120 Heidelberg, Germany; 3 Unité de Pathogénie Microbienne Moléculaire, U389 INSERM Institut Pasteur; 28, Rue du Docteur Roux, 75724 Paris Cedex 15, France; 4 Unité de Embryologie Moléculaire, Institut Pasteur; 25, Rue du Docteur Roux, 75724 Paris Cedex 15, France; 5 Current address: Department of Cell Biology, University of Cordoba, 14014-Cordoba, Spain; 6 Département de Biologie Cellulaire, Institut Jacques Monod, CNRS, Universités Paris 6 and 7, 75251 Paris Cedex 05, France; 7 Plate-Forme d'Imagerie Dynamique (PFID), Département de Biologie Cellulaire et Infection, Institut Pasteur; 25, Rue du Docteur Roux, 75724 Paris Cedex 15, France

## Abstract

**Background:**

There exists abundant molecular and ultra-structural evidence to suggest that cytoplasmic actin can physically interact with the nuclear envelope (NE) membrane system. However, this interaction has yet to be characterised in living interphase cells.

**Results:**

Using a fluorescent conjugate of the actin binding drug cytochalasin D (CD-BODIPY) we provide evidence that polymerising actin accumulates in vicinity to the NE. In addition, both transiently expressed fluorescent actin and cytoplasmic micro-injection of fluorescent actin resulted in accumulation of actin at the NE-membrane. Consistent with the idea that the cytoplasmic phase of NE-membranes can support this novel pool of perinuclear actin polymerisation we show that isolated, intact, differentiated primary hepatocyte nuclei support actin polymerisation *in vitro*. Further this phenomenon was inhibited by treatments hindering steric access to outer-nuclear-membrane proteins (e.g. wheat germ agglutinin, anti-nesprin and anti-nucleoporin antibodies).

**Conclusion:**

We conclude that actin polymerisation occurs around interphase nuclei of living cells at the cytoplasmic phase of NE-membranes.

## Background

The nuclear envelope (NE) consists of two adjacent membranes, the inner (INM) and the outer nuclear membrane (ONM). The ONM is functionally connected to the endoplasmic-reticulum (ER) and contains numerous ribosomes, while the INM contains a unique set of transmembrane proteins and maintains close contact with chromatin in the nuclear matrix through the nuclear lamina network [1,2]. The ONM/ER comprising a single continuous membrane system is connected to the INM through nuclear pore complexes (NPC), which constitute the unique gateway for macromolecular transport across the nuclear-cytoplasmic boundary [3,4]. The cytoskeletal protein actin may play a role in NE function. This view has received support from recent evidence suggesting that the NE-membrane system is physically connected to the cytoplasmic microfilament network. For example, ONM proteins containing giant spectrin repeats or SUN (for Sad1p, UNC-84 homology) domains are involved in nuclear anchorage and migration, probably via actin filament interactions [5,6]. In metazoan cells, the integral ONM protein *nesprin *(member of the Syne/ANC-1 protein family) contains an α-actinin like actin-binding domain potentially capable to link the NE-membrane to the cytoplasmic actin cytoskeleton [7].

Actin is a highly conserved cytoskeletal protein that is distributed in a dynamic equilibrium between the ~42 kD monomeric (*G-actin*) and the filamentous polymerised (*F-actin*) forms. Actin's endogenous, polymeric properties are preserved *in vitro *and have been extensively characterised [8,9]. Polymerisation occurs at the so-called critical concentration, where non-covalent interactions overcome a rate limiting step yielding trimeric nucleation complexes to which monomers associate spontaneously and seed filament growth [10]. Inside living cells, the non-steady-state dynamics of actin polymerisation are highly regulated by an array of actin binding proteins (for review see [11-13]). Actin binding proteins regulate changes in actin's polymerisation state within functional sub-cellular domains or compartments allowing for actin's role in a variety of cellular processes as diverse as signal transduction and cell motility. Importantly, the ONM protein nesprin enhances actin polymerisation rates, shortens filament elongation times, and increases filament bundling *in vitro*. Therefore, it has been hypothesised that nesprin (or other cytoskeletal microfilament binding ONM proteins) can recruit actin to the cytoplasmic ONM interface [7].

Previously, ultra-structural studies using electron microscopy in fixed material have demonstrated "filament-like" networks continuous with the NE-membrane and NPC [14,15]. Moreover, light microscopic studies have reported a fine perinuclear "shell" of actin filaments around the nuclei of fixed cultured cells [16]. In the current study we visualised actin polymerisation in proximity to the NE-membrane inside living cells. This approach has been technically challenging, in part because the compartmentalised gradient is expected to be small (compared with large backgrounds coming from actin involved in other processes). We overcame these difficulties, using a variety of fluorescent probes for actin and confocal fluorescence imaging methods. We report perinuclear actin inside intact living cells during interphase, and we provide evidence that perinuclear actin polymerisation involves binding at the ONM of the NE-membrane.

## Results

### Cytochalasin D binds NE-membranes

**Figure 1:**
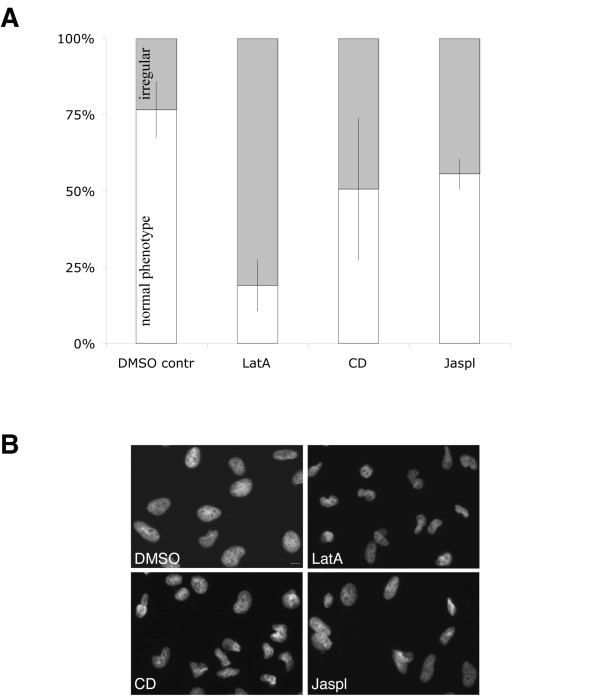
**Treatment of cells with actin interfering drugs**. HeLa cells were treated for 30 min at 37°C with the actin-binding drugs LatA (1μM), CD (1μM) Jaspl (200 nM), or vehicle (DMSO) and then stained with Hoechst 33342. Nuclear shape phenotypes were distinguished ("normal/round" or "irregular" nuclei) and counted by widefield microscopy. (**A**) The bar graph shows the percentage of normal nuclei (white) versus irregular nuclei (grey) measured from two independent experiments (see methods). (**B**) The panel shows an example of normal nuclei (after treatment with vehicle) compared with irregular nuclei following drug treatments. Scale bar: 5μm

Actin binding drugs alter the gross shape of cell nuclei in eukaryotic cells during interphase. In untreated HeLa cells (or those treated with DMSO vehicle) we noticed that the nuclear shape (revealed by Hoechst staining) was in general characterised by a smooth, round shape, whereas ~20% of the nuclei were irregular in shape. Irregular nuclei were characterised by asymmetric abnormalities including lobes and/or invaginations. In HeLa cells treated for thirty minutes with the actin binding drugs lantruculin A (*LatA*), cytochalasin D (*CD*) or jasplakinolide (*Jaspl*) a significant two-to four-fold increase in the percentage of cells displaying NE-membrane irregularities was observed (fig.1A; see also fig.1B for an example of cells after the respective treatment). Of the drugs tested, CD was of particular interest. This fungal metabolite binds the free-barbed-end of actin filaments and thereby blocks actin polymerisation. The effect of CD on nuclear shape raised the possibility that the NE-membrane is directly a target for the drug. To address this question, we profited from the availability of a fluorescent CD conjugate: CD-BODIPY *TMR *(tetramethylrhodamine) to detect CD binding-sites at the NE-membrane using fluorescence microscopy.

**Figure 2:**
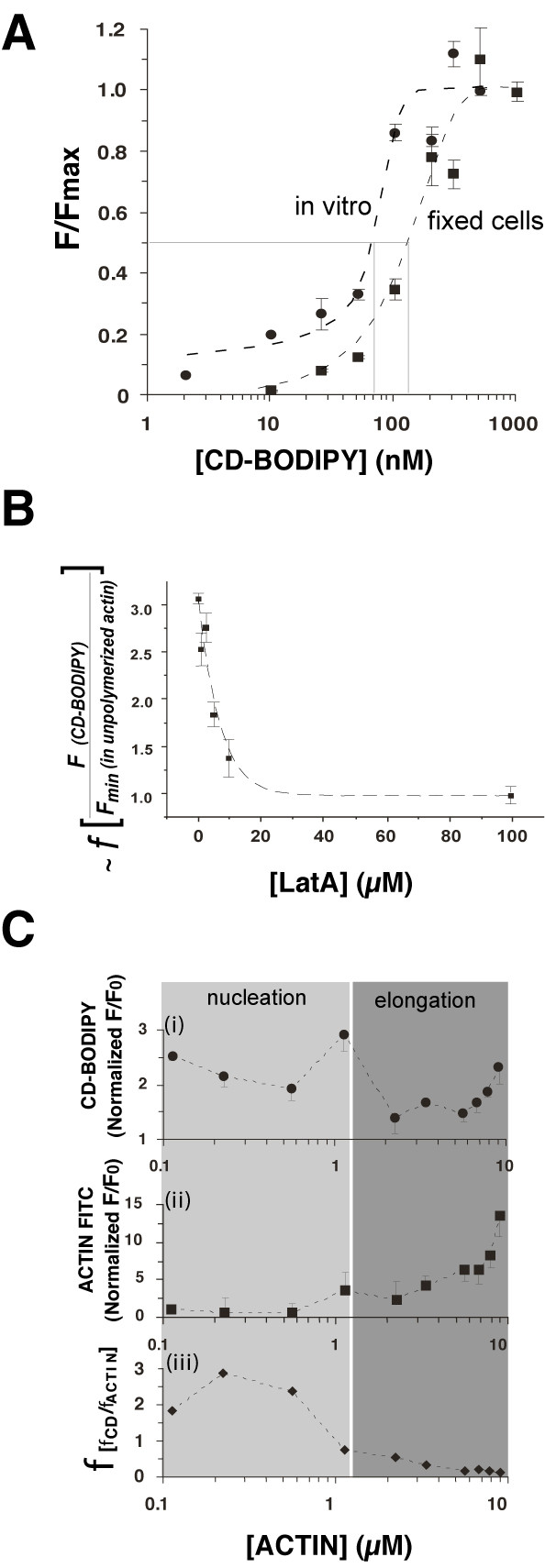
**CD-BODIPY binds free-barbed-end actin protomers *in vitro***. (**A**) Concentration dependency of CD-BODIPY fluorescence in polymerised actin solutions (*heavy dashed line, filled circles*). The drug's dose dependent fluorescence in fixed cells (HEK293) is also shown (*filled squares, fine dashed line*). All data are normalised to fluorescence maxima at 1μM CD-BODIPY. (**B**) Measurement of the concentration dependent effect of LatA on CD-BODIPY fluorescence (50 nM) in the presence of polymerised actin. Data points are normalised to the fluorescence measured in unpolymerised samples containing 10μM (ATP. actin). Note that in the absence of LatA, CD-BODIPY fluorescence in the presence of polymerised actin is three-fold higher than that measured in unpolymerised actin solutions. (**C**) *(i) *fluorescence of 50 nM CD-BODIPY in the presence of increasing [actin] in the presence of ATP (*circles*); *(ii) *corresponding FITC-actin fluorescence (*squares*) measured from the same sample fields. Fluorescence data are normalised to the average signals measured in unpolymerised conditions ([actin]) = 8.8μM). Graph *(iii) *shows the ratio of normalised CD-BODIPY fluorescence to normalized FITC-actin fluorescence. The border between the two shaded areas indicating conditions favouring short (*light grey shading*) or long (*dark grey*) actin polymers defines the critical concentration expected for actin polymerisation in the presence of 50 nM CD [17]. In all graphs vertical bars show S.E.M. from triplicate samples.

Before performing live cell *in situ *experiments we examined the actin binding characteristics of CD-BODIPY-TMR *in vitro *and in fixed cells. CD-BODIPY fluorescence was measured *in vitro *at various concentrations (2–1000 nM; fig.2A) in solutions containing fully polymerised rabbit skeletal muscle actin at 10 μM (a concentration far above the critical concentration for polymerisation wherein ~100% of available actin is in the polymeric form [17]). Under these *in vitro *conditions a concentration dependent, sigmoid shaped increase in CD-BODIPY fluorescence was observed, which closely resembled its behaviour when added to fixed cells at the same concentration (fig.2A). Both *in vitro *and in fixed cells these results suggest that the drug bound a saturable actin-binding site in the absence of dynamic actin filament turnover. In a second, *in vitro *assay we tested CD-BODIPY's fluorescence characteristics in the presence of polymerised actin and lantruculin A (LatA). LatA specifically binds G-actin (1:1), but not F-actin, and the resulting reduction in available monomers, shifts the dynamic steady-state equilibrium for actin filament formation towards depolymerisation [18]. In the presence of a constant concentration of actin (~10μM) and CD-BODIPY (50 nM), the effect of increasing concentrations of LatA was a net reduction in CD-BODIPY fluorescence (fig.2B). This effect was linear at drug concentrations between 1–10μM, and half-maximal at ~5μM, reflecting the predicted stoichiometry given the concentration of actin present. Since only the concentration of LatA was modified in this assay, the three-fold change in CD-BODIPY fluorescence could be explained by fluorescence enhancement of the drug's tetramethylrhodamine (TMR) moiety, upon binding to free-barbed-end protomers. Fluorescent enhancement resulting from TMR-drug binding to actin has been previously reported for *TMR-phalloidin*, and attributed to the sensitivity of TMR-X moiety in proximity to the hydrophobic environment of actin protomers [19,20]. This increase in fluorescence is a favourable property for the drug's use as a probe because it enhances specific signal detection. In light of this fact, we next examined CD-BODIPY fluorescence in a pure solution of actin around the critical concentration. Accordingly, we found that CD-BODIPY (50 nM) fluorescence peaked in pure solutions of actin at the critical concentration (~1.1μM; [17]) as shown in fig.2C(i)). Furthermore, relative to the total amount of actin present (fig.2C(ii)), CD-BODIPY fluorescence was diminished at levels at and above the critical concentration (fig.2C(iii)). In contrast, below the critical concentration the CD-BODIPY fluorescence relative to total actin present was at its highest levels, consistent with the view that free-barbed-end protomers and tri-meric nucleation complexes were most abundant at these concentrations (fig.2C(iii)). These in vitro results strongly suggested CD-BODIPY's utility as a fluorescent probe to detect CD binding sites *in situ*.

**Figure 3:**
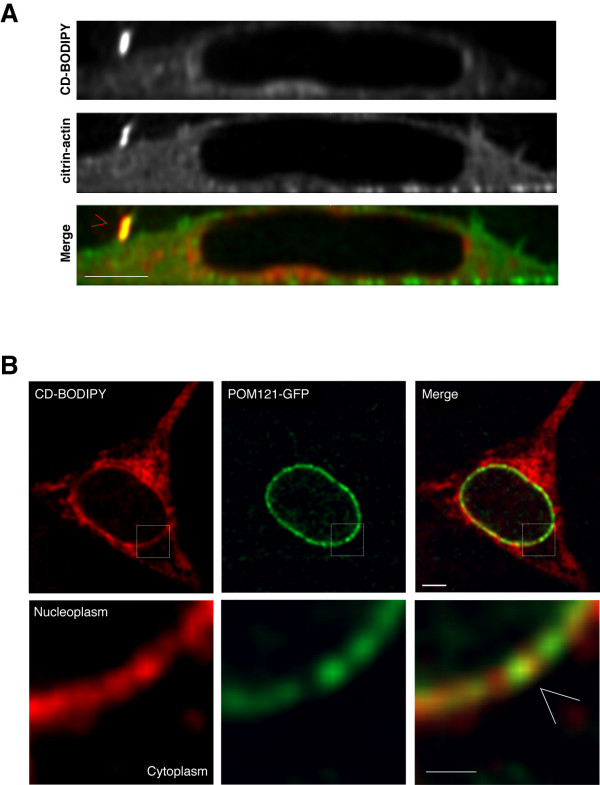
**Compartmentalisation of CD-BODIPY inside living cells**. (**A**) Shows a YZ optical section through a 3D confocal stack recorded from a single living HeLa expressing citrin-actin and stained with 50 nM CD-BODIPY for 20 minutes. The top and middle rows show corresponding CD-BODIPY and citrin-actin channels respectively, whereas the bottom shows the corresponding overlay image. Note the co-compartmentalisation of CD-BODIPY and the citrin-actin at a microspike protruding from the top of the cell (*red cursor*). Scale bar 5μm. (**B**) Single confocal optical section through a H-PomGFP (*green*) living cell co-stained with 50 nM CD-BODIPY (*red*) and the corresponding colour overlay image. Note strong accumulation of CD-BODIPY at the perinuclear proximity and the presence co-incident with NE-membrane labelling. Bottom panels show detail from selected region (*white rectangle*) highlighting where in this representative example NE-membrane signals were clearly distinguished from cytoplasmic staining revealing a clear view of compartmentalisation between CD-BODIPY and POM121 (*white cursor*). Scale bars: top row: 5μm; bottom row: 2μm.

For *in situ *visualisation inside living cells we used CD-BODIPY at very low concentrations (5–50 nM) relative to the drug's expected affinity for actin. This provided sufficiently strong staining allowing for signal detection, without interfering with actin polymerisation [21]. Within a few minutes after adding the fluorescent drug to the incubation medium we observed CD-BODIPY accumulation into plasma membrane *microspikes *that were co-labelled with fluorescent actin expressed in the same cell (fig.3A). This observation was consistent with the expected binding properties of CD-BODIPY, because these tiny sub-cellular compartments are characteristically concentrated with polymerising actin [22] and are highly sensitive to disruption by cytochalasin D. In a separate series of experiments, HeLa cells stably expressing the transmembrane nuclear pore complex (NPC) protein POM121 conjugated to the green fluorescent protein (GFP) [23] (H-PomGFP cells) were co-stained with CD-BODIPY. In these experiments, CD-BODIPY was observed to accumulate strongly in the deep perinuclear cytoplasm and across NE-membranes in almost all cells analysed. Based upon the distribution of the POM121 NPC signal, CD-BODIPY was localised to the NE-membrane however we did not detect a strict signal co-localisation pattern (fig.3B). Indeed across small stretches of the NE-membrane we discerned both positive and negative signal correlation among adjacent, near-sub-resolution regions of interest between CD-BODIPY and POM121-GFP fluorescence (fig.3B, lower panel, white arrow). Thus, CD-BODIPY labelling formed a discontinuous pattern comprising punctuate accumulation along the nuclear membrane. This staining pattern suggests strongly that CD-BODIPY binds directly at the NE-membrane, but is not strictly co-localised with NPCs.

### Actin accumulates at the cytoplasmic phase of NE-membranes

**Figure 4:**
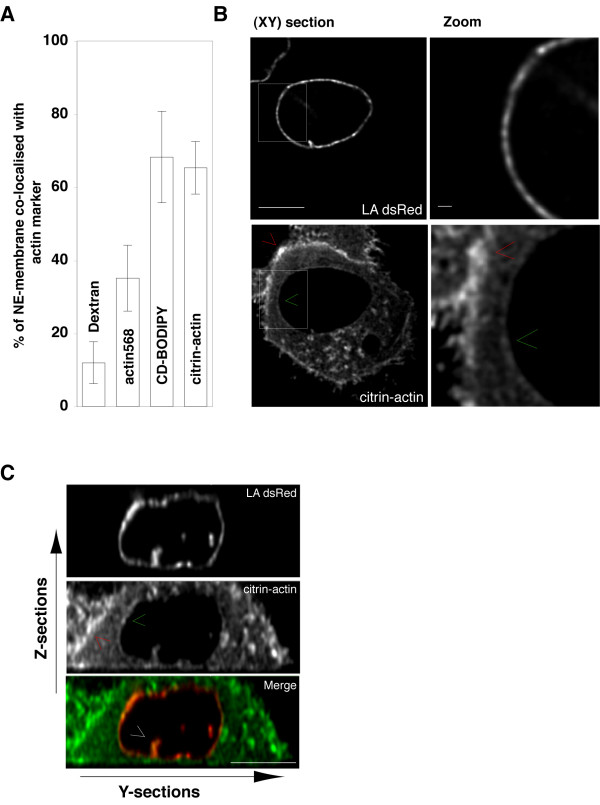
**Perinuclear actin revealed with a range of actin staining methods**. (**A**) Relative measure of actin accumulation at the NE inside living cells. Average values (n = 5 cells in each case) were calculated (see methods) from normalised intensity values mapped in three-dimensional image stacks recorded from living cells labelled using various protocols to reveal actin and NE-membranes. Results show averages of the percentage of NE-membrane label (either POM121-GFP or dsRed-laminA) that was *not* distinguishable in spatial coordinates (given resolution limits) from actin labelling probes (actin568, CD-BODIPY, citrin-actin). Averaged results (n= 5 cells) were compared (P < 0.01; *student t-test*) with those calculated from samples labelled by cytoplasmic micro-injection of dextran-rhodamine inside cells expressing POM121-GFP (**B**) Spinning disk confocal image of single living HeLa cell expressing lamin A dsRed (upper panels) and citrin-actin (lower panels). The XY optical section shows accumulation of cortical actin at the plasma membrane (*red *cursor). The green cursor indicates the faint perinuclear actin accumulation. The right panels show detail from the regions of interest indicated by the white-line dashed boxes in the left-hand panels. Scale bars: left panels 7μm; right panels 0.5μm. (**C**) Shows the YZ section view reconstructed from the same 3D through-stack dataset as (B) above. The red and green cursors show cortical and perinuclear actin accumulation as for (B). Note also the presence of an interphase nuclear invagination where dsRed-LaminA and citrin-actin both intrude upon the nuclear volume (white arrowhead). Scale bars: 7μm.

In H-PomGFP cells we observed ~70% of NE-membrane POM121-GFP signal was spatially coincident with CD-BODIPY signal, a value significantly greater (*P *< 0.01; *n *= 5) than that measured from control experiments (dextran injection; see methods; fig.4A). This suggests the presence of free-barbed-end actin-filaments in close proximity to the NE-membrane. To rule out the possibility that this accumulation of NE-membrane associated actin was due to treatment with CD-BODIPY, we repeated the same imaging protocol and quantification method using H-PomGFP cells labelled by cytoplasmic microinjection of fluorescent actin (Alexa568). Within minutes of microinjection into cell cytoplasm, the fluorescent (Alexa568) actin was detected in a ring pattern around cell nuclei similar to that observed for CD-BODIPY. A series of experiments revealed ~37% of green POM121-GFP signal was coincident with cytoplasmically micro-injected actin (fig.4A) a value still significantly (*P *< 0.01; *n *= 5) higher than that measured in systematic control experiments. We next investigated the sub-cellular distribution of *citrin-actin *[24] expressed in a HeLa cell line stably expressing red fluorescent nuclear lamin A (LA-dsRed). Citrin-actin accumulated at the cell cortex (fig.4B and 4C; red arrows), and in a faint, but distinct ring-like pattern close to the NE-membranes (fig.4B and 4C; green arrows). Delineated by the nuclear lamina labelling, the perinuclear actin signal was observed in both the reconstructed XY (fig.4B) and orthogonal YZ (fig.4C; red arrow) section views. In these reconstructions the fluorescence intensity of perinuclear actin appeared brighter than the actin fluorescence detected in the nearby cytoplasm (fig.4B right panel). The three-dimensional analysis of signal coincidence revealed that about 65% of LA-dsRed nuclear lamina signal was significantly (*P *< 0.01; *n *= 5) co-incident with citrin-actin (fig.4A).

Further, we observed that the distribution of micro-injected fluorescent actin in 3D rendered reconstruction from through stack image series comprises a punctuate discontinuous labelling across the NE-membrane [see Additional file 1]. A pattern strongly analogous to that observed using cells stained with CD-BODIPY and POM121-GFP [see Additional file 2].

**Figure 5:**
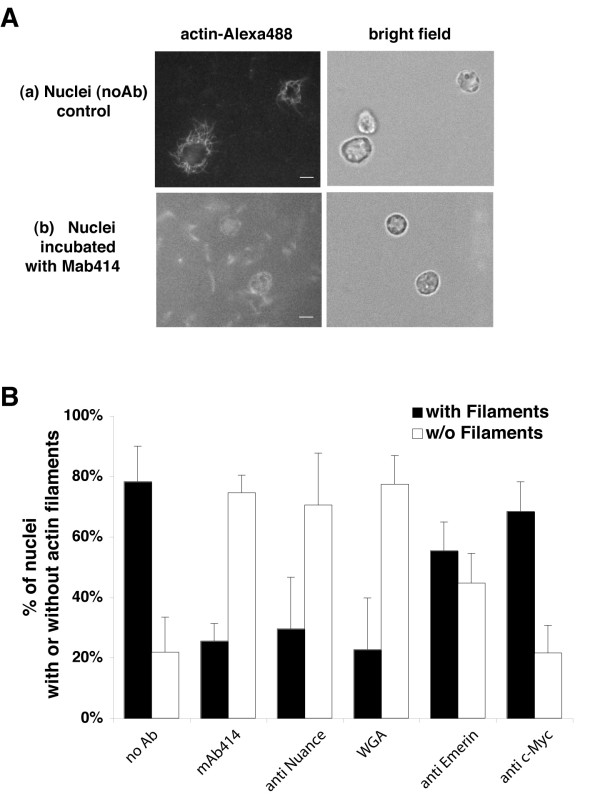
**Actin polymerisation around isolated, differentiated, intact nuclei *in vitro***. (**A**) Isolated rat liver nuclei were incubated for 1 h at 37°C with 500 nM actin Alexa488 under polymerising conditions and then imaged by fluorescence microscopy. (a): Fluorescent actin-Alexa488 assembled around isolated whole intact nuclei (left panel). The nuclei were identified by DIC imaging (right panel). (b) Shows representative examples of nuclei that were pre-incubated with mAb414 (see methods) and subsequently subjected to the actin polymerisation assay. After this treatment, the nuclei did not polymerise actin at their cytoplasmic surface (fluorescent image (left panel) has been contrast enhanced in order to reveal background staining of nuclei as well as dim residual actin filaments) (left panel: actin Alexa488 image and DIC on the right panel). (**B**) The effect of various antibodies on the polymerisation of actin around isolated nuclei. Note how both mAb414, and antibodies against Nuance or the non-specific nuclear transport inhibitor wheatgerm-agglutinin WGA significantly reduced actin polymerisation. By contrast anti-Emerin had no significant effect compared with anti-c-Myc (three independent experiments 50 nuclei/condition).

The live cell imaging experiments suggested that cytoplasmically injected actin rapidly accumulated at the NE-membrane. Therefore, we presume that freely diffusing actin in the cytoplasm had direct access to the perinuclear actin pool. We therefore used an *in vitro *actin polymerisation assay to examine the gross formation of actin polymers around highly purified nuclei, freshly isolated from rat liver hepatocytes. Intact hepatocyte nuclei were incubated in the presence of actin under conditions favouring actin polymerisation. Under these conditions we found that fluorescent actin polymerised in a halo around isolated intact nuclei. Testing several actin concentrations we observed this phenomenon at concentrations as low as 100 nM actin (~10% of the expected critical concentration; data not shown). In the presence of only 500 nM fluorescent actin (just half the critical concentration expected for *in vitro *conditions) we observed polymerised, fluorescent actin microfilament bundles in a halo around approximately 80% of individual nuclei (fig.5A(a)). Pre-incubation of intact nuclei with antibody against FXFG-repeat containing nucleoporins (*mAb414*); and non-nucleoporin (anti-nuance, antibody targeting ONM non-nucleoporin nesprin/nuance) both resulted in a decrease in the number of nuclei displaying actin filaments (fig.5B). Furthermore, the non-specific nuclear transport inhibitor wheat germ-agglutinin (WGA) also significantly reduced the percentage of nuclear actin halos (Mann-Whitney test: *P *< 0.05). By contrast, pre-incubation with antibodies to INM proteins (anti-Emerin, a protein epitope not accessible at the surface of intact nuclei) had no significant effect compared with both negative (no added antibody) and positive (anti-c-Myc) control conditions (Mann-Whitney test: *P *> 0.05). These experiments strongly suggest that actin polymerisation is localised to the cytoplasmic phase of intact isolated NE-membranes *in vitro*.

## Discussion

Previous studies using conventional approaches in fixed cells have described evidence for perinuclear actin in a variety of cell types including giant polyploid silk gland epithelial cells [25], insect cells [26], amobea [27] and fungi [28]. In vertebrate tissue cells a perinuclear actin basket, revealed by phalloidin has also been reported [29,30]. However, these studies were limited to a visualisation of actin after cell fixation, and lacked information on actin polymerisation, *in situ*, ie inside living cells. In the current study, we provide evidence for perinuclear actin polymerisation using spinning disk confocal microscopy. Our results show that inside intact living cells actin polymerises at NE-membranes, in a manner involving free-barbed-end protomers and through yet unidentified molecular components at the cytoplasmic face of the NE-membrane.

Actin is one of the most abundant proteins in eukaryotic cells and this exacerbates detection of specifically localised subcellular compartments or gradients, because the vast majority of measured signal is derived from actin bound in very bright structures (e.g.: stress fibers) or G-actin. Evidently, this is less problematic for studies investigating actin polymerisation in more accessible regions for imaging e.g. actin polymerisation at the leading edge of the plasma membrane [8,31-33]. By contrast, in the current study our interest was focused on perinuclear actin. To visualise actin in the perinuclear cytoplasm we used spinning disk confocal microscopy in order to maximise three-dimensional resolution. With this approach it was striking that a nuclear actin ring was observed using several types of probes for visualisation, but was not readily observed using conventional wide-field imaging. The need for spinning disk confocal microscopy followed by deconvolution can explain why the perinuclear actin ring has until now not been described inside living cells.

Using confocal imaging our results reveal that cytoplasmic micro-injection led, within minutes, to accumulation of fluorescent actin at the NE-membrane. This rapid accumulation of micro-injected actin fits with the idea that the perinuclear pool of actin and cytoplasmic actin are in dynamic equilibrium. One mechanism for cytoplasmic G-actin to interact with the perinuclear actin pool could be that the latter concentrates actin binding sites that present free-barbed-ends to which free cytoplasmic G-actin may further associate. Certainly, our results using CD-BODIPY are highly consistent with this view. We have shown that CD-BODIPY that binds free-barbed-end actin with high affinity *in vitro*, yields a labelling pattern coincident with the NE-membrane *in situ*. Pertinently, the NE-membrane associated CD-BODIPY signal was observed to extend into the perinuclear cytoplasm, but never into the nuclear matrix, suggesting that the signal is continuous with the cytoplasmic actin cytoskeleton. However, given the sensitivity and resolution limits of optical microscopy this does not rule out the possibility that actin polymerisation could be physically located at the inner-face of the NE-membrane.

A recent study has shown that microinjected actin is localised to the NE-membrane of starfish oocytes, in a ring pattern around the nucleus [34] analogous to our findings presented in the current work. This study showed that a transient burst of actin polymerisation occurs upon NE-membrane fenestration marking the beginning of nuclear envelope breakdown (NEBD), and M-phase progression. Using ultra-structural electron microscopy Lenard et al. localised actin polymerisation to the inner face of the NE-membrane in accordance with their model. Our current study using high-resolution multidimensional light microscopy raised the same question: i.e. whether polymerisation occurs at the inner-(nuclear matrix) or outer-(cytoplasmic) face of the NE-membrane. By contrast to the actin polymerisation upon NEBD, our results suggest that perinuclear actin polymerisation in interphase nuclei of metazoan cells is driven from physical interactions at the *cytoplasmic *side of the NE-membrane. *In vitro*, we observed long actin filaments propagating outwards from intact nuclei isolated from differentiated rat liver cells, and incubated under conditions favouring actin polymerisation. Furthermore, nuclei were intact and apparently impermeable (in the absence of GTP) to actin because we never observed fluorescent actin inside the nuclear matrix. Additional argument supporting the view that actin binds the cytoplasmic face of isolated nuclei was derived from our results indicating that actin polymerisation was strongly diminished in the presence of antibodies (or lectin) targeting ONM proteins, but not INM. Both antibody (mAb414) and lectin targeting ONM nucleoporins inhibited actin polymerisation as strongly as anti-nesprin (a non-nucleoporin target). Therefore we hypothesise that these effects were due to molecular steric hindrance of a cytoplasmic ONM actin binding site closely juxtaposed with nucleoporins and/or non-nucleoporins. This interpretation is also consistent with our observation that no actin labelling method yielded precise co-localisation with the nucleoporin POM121. Despite the uncertainty concerning whether nucleoporins and/or non-nucleoporins provide the actin binding site, our results do demonstrate that intact interphase nuclei support actin polymerisation at the cytoplasmic face of ONM. Interestingly, previous studies on isolated patch-clamped nuclei have provided evidence that NPC ionic permeability measured by electrophysiology can be modulated by actin added to the ionic bathing solution suggesting that the effect involved cytoplasmic ONM interactions [35]. Moreover, a role for NPC-associated cytoplasmic cytoskeleton filaments in regulation of NPC transport functions has been postulated [14]. In either case, taken together with our current results, these hypotheses provide for a plausible functional impact linking the signalling milieu of the cytoplasmic actin cytoskeleton with NPC structure and function.

## Conclusion

A dynamic pool of actin filaments is linked to the cytoplasmic face of nuclei in metazoan cells during interphase. Further studies will now aim at the identification of the factor (or factors) responsible for targeting actin polymerisation to the NE-membrane, and precisely defining the functional implications therein.

## Methods

### Cell lines and cell culture

HeLa (ATCC) were cultured in complete DMEM (DMEM Glutamax (Gibco BRL), 10% FCS, 100 U ml^-1 ^streptomycin, 100 μg ml^-1 ^penicillin) at 37°C; 5% CO_2_. H-PomGFP cells: nuclear pore labelling in HeLa cells was achieved by transfecting cells (*Fugene6*, Boehringer-Mannheim) with a plasmid containing POM121-GFP (kind gift of E. Hallberg, University College, Huddinge). Citrin-actin (kind gift of A. Hoppe, University of Michigan) was expressed where stated in HeLa cells using the same transfection method. Citrin-actin is a fusion construct of actin with an improved YFP variant [36]; the plasmid used in the current study has been described elsewhere [24].

### Constructing HeLa cells stably expressing LaminA-dsRed

Prelamin A cDNA cloned into pSVK3 plasmid was amplified by PCR, with an *Eco*RI restriction site engineered at the 5' end of the sense primer (5'-GCG AAT TCT ATG GAG ACC CCG TCC CAG CGG-3') and a *Kpn*I restriction site engineered at the 5' end of the antisense primer (5'-GC GGT ACC TTA CAT GAT GCT GCA GTT CTG-3') (as described in [37]). The reaction product was digested with *Eco*RI and *Kpn*I and ligated in frame into purified, linearised pDsRed-C1 (Clontech, Inc, Palo Alto, *CA*) after digestion with *Eco*RI and *Kpn*I. HeLa cells cultured to 80% confluency were transfected in chamber slides with Lipofectamine PLUS (Life Technologies, Inc., Gaithersburg, USA.), following the manufacturer's instructions. Stable transfected cells were selected in the presence of antibiotic (1 mg ml^-1 ^G418; Gibco Life Technologies Ltd). Selection of dsRed-positive cells was performed with FACS (fluorescence-activated cell sorter) analysis.

### Actin-binding drug treatment of HeLa cells

HeLa cells were grown on cover-slides and treated for 30 minutes with cytochalasin D 1μM (Sigma), latrunculin A 1μM (Molecular Probes) or jasplakinolide 200 nM (Molecular Probes) at 37°C. Then cells were washed with PBS, fixed for 5 minutes with 3%PFA at RT, stained with Hoechst 33342 (Sigma-Aldrich) (10 min, 0.001 mg ml^-1^) and mounted on slides. The nuclei were imaged with a widefield microscope (see below) and nuclear shape was evaluated and counted in two independent experiments. For each experimental condition at least 150 nuclei from at least ten microscopic fields were counted.

### *In vitro *actin polymerisation assay on isolated rat liver nuclei

Nuclei were purified from rat liver [38]: Rats were asphyxiated with CO_2_, and were then decapitated. The livers were isolated, placed into ice-cold low sucrose buffer (0.25 M sucrose, 50 mM Tris-HCl pH 7.5, 25 mM KCl, 5 mM MgCl_2_, 1 mM DTT, 0.5 M PMSF and 1 μg ml^-1 ^leupeptin/aprotinin/pepstatin) and minced. Then, 1 volume of minced liver and 2 volumes of the same buffer were homogenised in a Potter-Elvehjem homogeniser (0.025 cm clearance) for 15 full strokes at 1,700 rpm. The homogenate was filtered through 4 layers of cheese cloth and centrifuged at 800 g for 15 minutes at 4°C. 1 volume of loose pellet was mixed with 2 volumes of high sucrose buffer (2.3 M sucrose, 50 mM Tris-HCl pH 7.5, 25 mM KCl, 5 mM MgCl_2_, 1 mM DTT, 0.5 M PMSF and 1 μg ml^-1 ^leupeptin/aprotinin/pepstatin) before overlaying 1 part of high sucrose buffer with 5 parts of the initial mixture and ultra-centrifuged at 28000 rpm for 1 hour at 4°C in a SW-28 rotor (Beckman, USA). The nuclei containing pellet was re-suspended in the low sucrose buffer and was again homogenised with 2 full strokes as described above. This homogenate was centrifuged at 800 g for 15 minutes at 4°C and the pellet contained the highly purified rat liver nuclei. These nuclei were re-suspended in the low sucrose buffer, and aliquots were stored at-80 °C. The number of nuclei was determined by measuring the OD_260 _(OD_260 _= 1 equals 3 × 10^6 ^nuclei). The quality and entireness of isolated nuclei was assured by light microscopy.

Fluorescent actin-Alexa488 (Molecular Probes) in G-buffer (0.2 mM ATP, 0.2 mM CaCl_2_, 1 mM DTT, 10 mM Tris-Hcl, pH 8) was polymerised at 0.5 μM by addition of a 1:9 dilution of 10x KMEI (500 mM KCl, 10 mM MgCl2, 10 mM EGTA, 100 mM imidazole, pH 7) in the presence of 1 unit of isolated rat liver nuclei (~3 × 10^6 ^nuclei). Isolated nuclei were pre-incubated with antibodies overnight at 4°C with continuous, gentle agitation. Antibodies used in this assay were mAb414 (Eurogentec, 1/1000), mAb anti-Nuance (kind gift of A. Noegel, University of Cologne; 1/10), anti-Emerin (kind gift of H. Hermann, DKFZ Heidelberg; 1/200) and mAb anti c-Myc (9E10 Santa-Cruz Biotechnologies, 1/1000). Additionally, we incorporated the non-specific nuclear transport inhibitor wheatgerm agglutinin (Invitrogen) in this assay. An aliquot of the reaction mix was sealed under a glass cover slip (coated with 2% cold water fish skin gelatin) on a microscope slide and then incubated for 1 hour at 37°C to allow time for actin polymerisation to occur. Finally, the number of nuclei displaying actin microfilaments was counted using a widefield microscope.

### Microinjection

Single cells were cytoplasmically microinjected with either 10 μM actin-Alexa568 (Molecular Probes) or 2.5 mg ml^-1 ^dextran-rhodamine 70 kDa (Molecular Probes) using a Femtojet/Injectman micromanipulator (Eppendorf, Hamburg, Germany).

### CD-BODIPY *in vitro *measurements

Purified, native and fluorescent (*FITC*) conjugated rabbit skeletal muscle actin (Cytoskeleton, Denver, *U.S.A*.) in 2 μl stock aliquots (10 mg ml^-1^; 222 μM) containing 2 mM Tris-HCl, 0.2 mM CaCl_2_, 0.2 mM ATP, 0.5 mM DTT, 2% sucrose, and 0.005% NaN_3 _(pH 8) were prepared at working dilutions (0.5–0.75 mg ml^-1^; 11–17 μM) by addition of low salt buffer (5 mM Tris-HCl, 0.2 mM Na-ATP, 0.5 mM DTT, 0.2 mM CaCl_2_, pH 8). For each experiment a single fresh actin stock was prepared. Drugs were added, yielding the final concentrations stated. Samples were vigorously mixed by pipetting, vortexed and then left on ice for 1 hour before centrifugation (100,000x g, 4°C, 30 minutes). Supernatants were added to 5x concentrated *high *salt polymerisation buffer giving final concentrations of 100 mM KCl and 10 mM Mg-ATP. After mixing, a 5 μl aliquot was sealed under a glass cover slip on a microscope slide and incubated at 37°C for at least 3 hours to allow actin to fully polymerise to a steady state [17] before semi-quantitative observation using fluorescence microscopy. Each experiment (triplicate samples) was performed at least twice yielding comparable results.

### Image acquisition, processing, and analysis

For live cell imaging, cells cultured on 35 mm glass bottom dishes (MatTek Corporation, USA) were exchanged into OptiMEM (Gibco BRL), and transfered to the microscope stage that was maintained at 37°C (Carl Zeiss "Tempcontrol 37–2 digital" incubator support). For experiments using CD-BODIPY, experimental medium was freshly prepared by serial dilution of a 1 mM stock solution (DMSO) to the stated final concentrations (2–50 nM). Facile and sufficient staining of cells was achieved by incubation of cells in this solution for 20 minutes before recordings began. In the presence of such very low concentrations, we found that cells could be maintained for several hours, or even days, without any deleterious effects. Using bright-field observation, we routinely ensured that observed cells were flat, adherent and healthy. Fluorescent imaging was performed using a high-speed spinning disk confocal system (UltraView RS Perkin-Elmer, USA) equipped for dual wavelength excitation (Kr-Ar laser; 488 nm and 568 nm) and axial z-stack sampling (PI objective piezo-drive). The system was built around a Carl Zeiss (Germany) Axiovert 200 microscope. Cells were visualised using a Hamamatsu (Japan) ERII camera (exposure time 200 ms) using a 100x *NeoFluor *NA 1.3 oil-immersion objective (Carl Zeiss). Fast axial (through-stack) sampling used a piezo-objective drive stepping at 200 nm throughout the cell volume.

For analysis, all axial image datasets were treated identically. First using geometric light reconstruction (deconvolution) based on a calculated point-spread-function, followed by a "maximum likelihood estimate" (MLE) iterative algorithm (Huygens software; Scientific Volume Imaging, Netherlands), in "Nipkow-disk" mode with parameters for the Yokagawa CSU-22 scan head. The deconvolved 3D through-stack results were then analysed for signal coincidence using the "Coloc" plug-in of *Imaris *(Bitplane, Switzerland). For all results we set a threshold based on the averaged background signal detected outside the region of interest in the channel used for NE-membrane detection (POM121-GFP, or dsRed-Lamin), such that the threshold identified a binary mask region delineating the NE-membrane signal. The second channel (Alexa568-actin, citrin-actin, or CD-BODIPY-TMR) was then similarly thresholded and masked. Quantification of signal co-incidence between two channels was then measured as a function of the percentage of pixels detected in the NE-membrane signal channel that were also positive for binary masked pixels derived from the actin dependent signal. This quantification process was applied to each image in any given stack, yielding an estimate of three dimensional signal co-incidence in a given cell. As a systematic control for this 3D signal coincidence we used exactly the same acquisition and analysis protocol except H-PomGFP cells were microinjected cytoplasmically with red fluorescent Alexa568-dextran. This provided us a relative measure of the microscope's ability to distinguish the NE-membrane (green) from diffusible non-specific cytoplasmic signal (red). On average (n = 5), throughout the imaged 3D volume we found that just ~12% of pixels containing specifically localised NE-membrane signal were spatially coincident with pixels also positive for red dextran signal. Given that the dimensions of the NE-membrane were below the optical resolution limits of the microscope, and that all two-color, three-dimensional analysis used the same imaging hardware/analysis this value served as a systematic threshold limit below which the NE-membrane and cytoplasmic signals could be considered "non-coincident".

Images of isolated intact nuclei for the *in vitro *actin polymerisation assay used a standard Zeiss Axiovert 200 M microscope equipped with a 40x objective, NA 1.3. The microscope was controlled by Till Vision (Till photonics, Germany) designed for fast live cell imaging and equipped with a xenon lamp, polychromator (*PolychromeIV) *for wavelength selection and a Till Photonic Imago QE CCD camera.

## Abbreviations

cytochalasin D: CD

cytochalasin D BODIPY: CD-BODIPY

HeLa POM121-GFP cells: H-PomGFP

Latrunculin A LatA

inner nuclear membrane: INM

Jasplakinolide Jaspl

nuclear envelope: NE

nuclear pore complex: NPC

outer nuclear membrane: ONM

tetramethylrhodamine TMR

## Competing interests

The author(s) declare that they have no competing interests.

## Authors' contributions

SM carried out microscopy and quantification studies. JE isolated and purified rat liver nuclei and contributed to writing of the paper. RVM carried out microinjection and analysis of these data. ED contributed LA dsRed cells. BDW and UN contributed to design of experiments and writing of the paper. SLS proposed the hypothesis, carried out the *in vitro *analysis of CD-BODIPY and conceived and designed experiments. SM and SLS wrote the paper. All authors read and approved the final manuscript.

## Supplementary Material

Additional File 1 The movies are viewable using QuickTime, which can be downloaded from Apple's QuickTime Site . **3D-image reconstructions of A) cytoplasmically injected fluorescent actin and B) CD-BODIPY visualised in living HeLa cells expressing fluorescent NPC (POM121-GFP). M1A **shows a maximum-intensity projection of three-dimensional dataset rendered from a confocal stack recording of an H-PomGFP cell subjected to cytoplasmic microinjection of fluorescent actin just minutes before the experiment. In this overlay movie projection the actin channel is pseudo-colored *red*, whereas POM121-GFP is pseudo-colored *green*. Click here for file

Additional File 2 The movies are viewable using QuickTime, which can be downloaded from Apple's QuickTime Site . **3D-image reconstructions of A) cytoplasmically injected fluorescent actin and B) CD-BODIPY visualised in living HeLa cells expressing fluorescent NPC (POM121-GFP). M1B **shows a maximum-intensity projection three-dimensional rendering of a confocal stack recorded from an H-PomGFP cell labelled with 50 nM CD-BODIPY. CD-BODIPY channel is pseudo-colored *red*, whereas POM121-GFP is pseudo-colored *green*. Note in both movies actin fluorescence distribution around the NE-membrane is punctate. Click here for file

## References

[B1] Gerace L, Burke B (1988). Functional organization of the nuclear envelope. Annu Rev Cell Biol.

[B2] Gant TM, Wilson KL (1997). Nuclear assembly. Annu Rev Cell Dev Biol.

[B3] Rout MP, Aitchison JD (2001). The nuclear pore complex as a transport machine. J Biol Chem.

[B4] Wente SR (2000). Gatekeepers of the nucleus. Science.

[B5] Reinsch S, Gonczy P (1998). Mechanisms of nuclear positioning. J Cell Sci.

[B6] Starr DA, Han M (2003). ANChors away: an actin based mechanism of nuclear positioning. J Cell Sci.

[B7] Zhen YY, Libotte T, Munck M, Noegel AA, Korenbaum E (2002). NUANCE, a giant protein connecting the nucleus and actin cytoskeleton. J Cell Sci.

[B8] Pollard TD, Borisy GG (2003). Cellular motility driven by assembly and disassembly of actin filaments. Cell.

[B9] Pollard TD, Cooper JA (1986). Actin and actin-binding proteins. A critical evaluation of mechanisms and functions. Annu Rev Biochem.

[B10] Kuhn JR, Pollard TD (2004). Real time measurements of actin filament polymerization by total internal reflection fluorescence microscopy. Biophys J.

[B11] Schoenenberger CA, Bischler N, Fahrenkrog B, Aebi U (2002). Actin's propensity for dynamic filament patterning. FEBS Lett.

[B12] Welch MD, Mullins RD (2002). Cellular control of actin nucleation. Annu Rev Cell Dev Biol.

[B13] Pollard TD, Blanchoin L, Mullins RD (2000). Molecular mechanisms controlling actin filament dynamics in nonmuscle cells. Annu Rev Biophys Biomol Struct.

[B14] Goldberg MW, Rutherford SA, Hughes M, Cotter LA, Bagley S, Kiseleva E, Allen TD, Clarke PR (2000). Ran alters nuclear pore complex conformation. J Mol Biol.

[B15] Kiseleva E, Drummond SP, Goldberg MW, Rutherford SA, Allen TD, Wilson KL (2004). Actin- and protein-4.1-containing filaments link nuclear pore complexes to subnuclear organelles in Xenopus oocyte nuclei. J Cell Sci.

[B16] Clubb BH, Locke M (1998). Peripheral nuclear matrix actin forms perinuclear shells. J Cell Biochem.

[B17] Carlier MF, Criquet P, Pantaloni D, Korn ED (1986). Interaction of cytochalasin D with actin filaments in the presence of ADP and ATP. J Biol Chem.

[B18] Coue M, Brenner SL, Spector I, Korn ED (1987). Inhibition of actin polymerization by latrunculin A. FEBS Lett.

[B19] Allen PG, Janmey PA (1994). Gelsolin displaces phalloidin from actin filaments. A new fluorescence method shows that both Ca2+ and Mg2+ affect the rate at which gelsolin severs F-actin. J Biol Chem.

[B20] Huang ZJ, Haugland RP, You WM (1992). Phallotoxin and actin binding assay by fluorescence enhancement. Anal Biochem.

[B21] Giannone G, Dubin-Thaler BJ, Dobereiner HG, Kieffer N, Bresnick AR, Sheetz MP (2004). Periodic lamellipodial contractions correlate with rearward actin waves. Cell.

[B22] Nobes CD, Hall A (1995). Rho, rac, and cdc42 GTPases regulate the assembly of multimolecular focal complexes associated with actin stress fibers, lamellipodia, and filopodia. Cell.

[B23] Soderqvist H, Imreh G, Kihlmark M, Linnman C, Ringertz N, Hallberg E (1997). Intracellular distribution of an integral nuclear pore membrane protein fused to green fluorescent protein--localization of a targeting domain. Eur J Biochem.

[B24] Hoppe AD, Swanson JA (2004). Cdc42, Rac1, and Rac2 display distinct patterns of activation during phagocytosis. Mol Biol Cell.

[B25] Henderson SC, Locke M (1992). A shell of F-actin surrounds the branched nuclei of silk gland cells. Cell Motil Cytoskel.

[B26] Jeun G, Locke M (1993). The timing of division in twin cell doublets. Tissue Cell.

[B27] Pomorski P, Grebecka L (1993). Is actin involved in the nuclear division in Amoeba proteus?. Cell Biol Int.

[B28] Butt TM, Heath IB (1988). The changing distribution of actin and nuclear behavior during the cell cycle of the mite-pathogenic fungus Neozygites sp. Eur J Cell Biol.

[B29] Clubb BH, Locke M (1996). F-actin forms transient perinuclear shells at the mitosis-interphase transition. Cell Motil Cytoskeleton.

[B30] Riparbelli MG, Dallai R, Callaini G (1993). The cortical actin cytoskeleton in a Dipteran embryo: analysis of the spatial reorganization of F-actin aggregates during the early nuclear division cycles. Biol Cell.

[B31] Ponti A, Machacek M, Gupton SL, Waterman-Storer CM, Danuser G (2004). Two distinct actin networks drive the protrusion of migrating cells. Science.

[B32] Schafer DA, Welch MD, Machesky LM, Bridgman PC, Meyer SM, Cooper JA (1998). Visualization and molecular analysis of actin assembly in living cells. J Cell Biol.

[B33] Anderson KI, Wang YL, Small JV (1996). Coordination of protrusion and translocation of the keratocyte involves rolling of the cell body. J Cell Biol.

[B34] Lenart P, Bacher CP, Daigle N, Hand AR, Eils R, Terasaki M, Ellenberg J (2005). A contractile nuclear actin network drives chromosome congression in oocytes. Nature.

[B35] Prat AG, Cantiello HF (1996). Nuclear ion channel activity is regulated by actin filaments. Am J Physiol.

[B36] Griesbeck O, Baird GS, Campbell RE, Zacharias DA, Tsien RY (2001). Reducing the environmental sensitivity of yellow fluorescent protein. Mechanism and applications. J Biol Chem.

[B37] Favreau C, Higuet D, Courvalin JC, Buendia B (2004). Expression of a mutant lamin A that causes Emery-Dreifuss muscular dystrophy inhibits in vitro differentiation of C2C12 myoblasts. Mol Cell Biol.

[B38] Blobel G, Potter VR (1966). Nuclei from rat liver: isolation method that combines purity with high yield. Science.

